# Notes and News

**Published:** 1894-01-06

**Authors:** 


### Notes and News.

Collected and Communicated.

A Conference has been held recently at Leeds between the honorary medical staffs of the three chief local medical charities and the Executive Committee of the Leeds Workpeople's Hospital Fund, to consider the abuse of the out-patient system. The conference revealed a most evident want of reform, but no very apparent resourcefulness or ability was displayed in dealing with the matter. It was deemed best, as a final conclusion, to revert to the old system of recommendation forms, the Workpeople's Hospital Committee acting as inquiring body.

The Convalescent Home for Men at Tyn-y-Coed, in connection with the Birmingham Hospital Saturday Fund, has proved of such great benefit that strenuous efforts are being made to establish a like institution for women. Several sites in Wales have been under consideration, the most favoured being Marl Hall, in the neighbourhood of Llanrhos. There is some historical interest attached to the place, which is charmingly situated, with extensive grounds attached. There is accommodation for from twenty-five to thirty beds.

It is only of late years that we have awakened to the urgent needs of epileptics. For a man or woman who is obliged to work to be stricken with this complaint is a tragedy which has few equals; they are barred from all means of livelihood through a scourge which, in many cases, appears only at rare intervals; but yet they are able to work, and willing, but employment cannot be given them. The German colony of Bethel at Bielefeld has been the outcome of a humane wish to help the victims of epilepsy to help themselves. It is an enterprise which has no unlikely rival in its planning and maintenance in other countries. It is neither an asylum nor a workhouse. The inmates have indoor work provided for them, each, so far as can be arranged, carrying on previous occupations. At the same time there is an established manufacture of bromide, which not only is used in the cases of the workers themselves, but has an export as well.

The Philadelphia Board of Health have refused to accept the diagnosis of "Heart failure" as a cause of death. That is to say, that any such cause being quoted will be followed by further inquiry. An American medical man, writing to a medical paper on the subject, infers that this decision is likely to encourage careful prescribing, as he considers that the use of certain strong drugs in favour in the treatment of cardiac diseases is responsible for the shortening, if they do not cause, many deaths.

The name of Stokes can never be forgotten in connection with the Birmingham Hospital Saturday movement. We now record further generosity on the part of the family in the same direction. The Misses Stokes have already expended upwards of £9,000 on the Convalescent Home in connection with the Saturday Fund at Tyn-y-Coed, and on the present occasion they are presenting the institution with the purchase money of the site, amounting to £2,000. Only one condition attaches to the gift, which is that no mortgage shall ever be entered into with respect to the estate.

A new dispensary has just been opened at Stourbridge. There was little ceremony on the occasion but in the evening a concert was given in aid of the Furnishing Fund. The old dispensary had become quite inadequate, when the management found themselves in possession of a sufficiently large sum, through legacies made by Mr. and Mrs. Cole, to commence building more suitable premises. The dispensary, built in the Tudor style, contains a large waiting hall, a good dispensary proper, and a consulting-room. The expenditure has been £1,700, whilst £150 has so far been subscribed for furnishing expenses.

American liquor legislation is occupying a great deal of public attention at the present time. This is but natural, seeing that in the drink question is involved much affecting the health, happiness, and prosperity of all nations. Legislation has always played a far larger rôle in the matter in America than in England, and the result has been watched with deep interest more especially in this country. Strong repressive measures have been an undoubted failure, and the Brooks Licensing Act passed in 1886 was found a necessary modification. This provided that the licensing should be left in the hands of the Courts of Quarter Sessions, and that a heavy licensing fee should in all cases be demanded. Although in Pennsylvania, where the new scheme first started, the result was excellent, the number of cases of drunkenness being reduced 72 per cent.; in Pittsburg, where it was also tried, the effect was not markedly good but retrograde. In Pennsylvania, it is stated, public opinion and the police strenuously upheld the regulations imposed, whilst in Pittsburg this was not the case, showing that legislature alone cannot be depended upon apart from public opinion. The various experiments in America should prove useful to our own lawmakers. They have been tried for a sufficiently long period to make it possible to draw deductions with reasonable accuracy, and will, it is hoped, aid the choice of the true principles for educating public opinion in the right direction towards removing the serious blot which rests against us as a nation.

